# Symptomatic venous thromboembolism following a hip fracture: Incidence and risk factors in 5,300 patients

**DOI:** 10.3109/17453670903448273

**Published:** 2009-12-04

**Authors:** Iain McNamara, Aman Sharma, Teresa Prevost, Martyn Parker

**Affiliations:** ^1^Department of Trauma and Orthopaedics, Peterborough Hospital; ^2^Centre for Applied Medical Statistics, University of Cambridge, UK

## Abstract

**Background and purpose** Venous thromboembolism (VTE) remains a substantial cause of morbidity and mortality following hip fracture. Previous work has not identified any risk factors associated with the type of hip fracture. We report the incidence of and risk factors for development of symptomatic VTE in patients following a hip fracture.

**Patients and methods** In this prospective study, we collected information on 5,300 consecutive patients who were admitted to a single unit with a hip fracture—in terms of their pre-admission status, details of any operation performed, and details of complications in the form of symptomatic venous thromboembolism. All patients received thromboprophylaxis with heparin.

**Results** The incidence of symptomatic VTE was 2.2% (95% CI: 1.8–2.6). 85% of these events occurred within 5 weeks of the fracture. The statistically significant risk factors for symptomatic VTE were better preoperative mobility, living in one's own home, high mental test score, high preoperative hemoglobin, inter-trochanteric fractures, and fixation with a dynamic hip screw. In multivariate analysis adjusting for sex and age, type of residence on admission, type of fracture, and hemoglobin values on admission remained independently significant.

**Interpretation** We found that the rate of symptomatic VTE using thromboprophylaxis with heparin was low but that there were a number of groups that were at a significantly higher risk of developing VTE. The patients who are particularly at risk appear to be those with a subtrochanteric or intertrochanteric hip fracture; here, the incidence of symptomatic VTE was twice that of intracapsular hip fractures.

## Introduction

Venous thrombosis is a substantial cause of morbidity and mortality in patients following hip fracture (Todd et al. 1995). Asymptomatic deep vein thrombosis (DVT) has been reported in up to 50% of all patients who sustain a hip fracture, with an incidence of fatal pulmonary embolus (PE) of up to 10% (Haake and Berkman 1989, Todd et al. 1995, Zahn et al. 1999). The incidence of asymptomatic thrombi will always be markedly higher than those that are clinically apparent.

The purpose of this study was to determine the incidence of symptomatic VTE in patients following hip fracture, from a single centre, to ascertain any pre-existing admission characteristics that might increase the risk, to examine the surgical risk factors, and to determine the time period over which the symptomatic VTE was diagnosed.

## Patients and methods

The details of all patients with a proximal femoral fracture presenting at a single institution (Peterborough District Hospital, U.K.) between January 1989 and January 2007 were recorded at the time of admission. The collection of data had been approved by the Hospital Review Board. Patients over the age of 16 years were included, including those with fractures secondary to tumors or with localized bone pathology—such as Paget's disease.

Preoperative data included the patient demographics, patient's residence preoperatively, mobility (including the use of walking aids), mental test score, and smoking status. In addition, the hemoglobin (g/L) on admission was recorded. The length of time from fracture to admission and from fracture to surgery was also documented.

Mobility was assessed using a mobility score rated from 0 to 9. A score of 9 represented full mobility without the use of walking aids, while a score of 0 signified that the patient was bed-bound (Parker and Palmer 1993). The mental test score was a series of 10 questions on recall, scored from 0 to 10 (Qureshi and Hodkinson 1974).

Operative details regarding the type of procedure performed, the length of surgery, and length of anesthesia were collected. Patients who were treated nonoperatively were excluded from any analysis involving operative timing. The type of anesthetic was documented, as was any co-morbidity (as assessed by ASA grade).

Fractures were classified into intracapsular, inter-trochanteric, and sub-trochanteric (Parker 2001). Intracapsular hip fractures were treated either with fixation with multiple screws or with a hemiarthroplasty. Extracapsular fractures were treated by internal fixation with a sliding hip screw or an intramedullary nail.

All symptomatic VTE cases were recorded, defined as any case of symptomatic deep vein thrombosis or pulmonary embolism. Deep vein thrombosis was defined as any thrombosis diagnosed by ultrasound, venography, or at autopsy. Pulmonary embolism was diagnosed by computer tomography pulmonary angiography (CTPA), nuclear medicine isotope scanning, or at autopsy. All patients presenting with symptoms of VTE were subject to investigation.

After discharge from hospital, at least 1 follow-up assessment was undertaken in a hip-fracture clinic after 6 weeks. Subsequent follow-up was by telephone with a final follow-up at 1 year after admission. Any patient who had been diagnosed with a VTE in the community was identified at the follow-up points.

All patients received thromboprophylaxis, which was given from the day of admission. From 1989 until 1992, unfractionated calcium heparin was used (5,000 units twice daily). In 1992, the thromboprophylaxis regime was changed to low-molecular-weight heparin (Enoxaparin, 40 mg once a day). The thromboprophylaxis regime was continued for 14 days after surgery. Graduated compression stockings and mechanical calf or foot pumps were not used. After surgery, all patients were mobilized as soon as possible.

### Statistics

Initially, the baseline characteristics of the group that presented with VTE were compared with the baseline characteristics of the group without VTE. The methods of analysis were chi-squared test for categorical variables, the Mann-Whitney test for variables involving length of time, and t-test for other continuous variables. Statistically significant risk factors for VTE were assessed together by multivariate analysis in a logistic regression model, adjusting for sex and age. Effects are presented as odds ratios, which approximate to relative risk due to the low incidence rates.

The VTE group was subdivided into the subcategories of deep vein thrombosis and pulmonary embolism, and compared with the group with no thrombosis using similar methods. All tests were two-sided, and were assessed both at the 5% level of significance—and also at the stricter 1% level. Analyses were done using SPSS version 12.0.1.

## Results

Between January 1989 and January 2007, 5,300 patients were admitted to our institution with a proximal femoral fracture. 86 patients were treated non-operatively, and they were therefore excluded from any analysis involving operative timing.

The average age of the patients was 80 (17–103) years and 78% were female. There were 117 venous thromboembolic complications (2.2%, 95% CI: 1.8–2.6). 79 patients (1.5%) were diagnosed with only a deep vein thrombosis and 38 (0.7%) presented with a pulmonary embolus (Table 1). There was a higher incidence of deep venous thrombosis in those patients who had a sliding hip-screw fixation and intramedullary nail (p = 0.01) (Table 2).

**Table 1.** Characteristics of the patients on admission related to the occurrence of thromboembolic complications (percentage with and without a thrombotic episode and its subcategories within the category of each characteristic)

	No thrombosis	Any thrombosis	Deep vein thrombosis	Pulmonary embolism
Total numbers	5,183	117	79	38
Sex				
Female	4,062 (98%)	97 (2.3%)	66 (1.6%)	31 (0.8%)
Male	1,121 (98%)	20 (1.8%)	13 (1.1%)	7 (0.6%)
p-value		0.3	0.3	0.7
Mean age (SD)	80 (11)	79 (12)	79 (11)	80 (9.9)
p-value		0.7	0.7	1
Admission mobility score (n = 5298) **^b^**				
8–9	1,466 (97%)	47 (3.1%)	34 (2.3%)	13 (0.9%)
4–7	1,834 (98%)	38 (2.0%)	27 (1.5%)	11 (0.6%)
0–3	1,881 (98%)	32 (1.7%)	18 (1.0%)	14 (0.7%)
p-value		0.01	0.008	0.6
Use of walking aids (n = 5293)				
None	2,833 (98%)	74 (2.5%)	52 (1.8%)	22 (0.8%)
Aids	2,230 (98%)	42 (1.8%)	27 (1.2%)	15 (0.7%)
Immobile	114 (100%)	0 (0%)	0 (0%)	0 (0%)
p-value		0.06	0.08	0.6
Residence on admission (n = 5300) **^b^**				
Own home	3,715 (97%)	99 (2.6%)	67 (1.8%)	32 (0.8%)
Institution	1,468 (99%)	18 (1.2%)	12 (0.8%)	6 (0.4%)
p-value		0.002	0.008	0.4
ASA grade				
1–2	1,750 (98%)	35 (2.0%)	24 (1.4%)	9 (0.5%)
3–5	3,331 (98%)	80 (2.3%)	54 (1.6%)	21 (0.6%)
p-value		0.4	0.6	0.7
Mental test score (n = 4849) **^b^**				
0–5	1,494 (99%)	19 (1.3%)	11 (0.7%)	8 (0.5%)
6–10	3,255 (98%)	85 (2.5%)	60 (1.8%)	25 (0.7%)
p-value		0.005	0.004	0.5
Smoking status (n = 4985)				
Non-smoker	4,267 (98%)	92 (2.1%)	62 (1.4%)	30 (0.7%)
Smoker	611 (98%)	15 (2.4%)	13 (2.1%)	2 (0.3%)
p-value		0.7	0.2	0.4
Mean hemoglobin on admission (g/L) (n = 5232) **^a^**	124	128	127	129
p-value		0.03	0.2	0.06
Type of fracture (n = 5300) **^b^**				
Intracapsular	2,917 (98%)	50 (1.7%)	30 (1.0%)	20 (0.7%)
Intertrochanteric	2,032 (97%)	63 (3.0%)	46 (2.2%)	17 (0.8%)
Subtrochanteric	180 (98%)	4 (2.2%)	3 (1.7%)	1 (0.6%)
p-value		0.008	0.002	0.8
Pathological fracture (n = 5046)				
No	4,916 (98%)	115 (2.3%)	77 (1.6%)	30 (0.6%)
Yes	22 (96%)	1 (4.3%)	1 (4.3%)	0 (0%)
p-value		1	0.3	1

**^a^** Risk factor for any thrombosis significant at the 5% level. **^b^** Risk factor for any thrombosis significant at the 1% level.

**Table 2.** Operation factors related to the occurrence of thromboembolic complications (percentage with and without a thrombotic episode and its subcategories within the category of each factor)

	No thrombosis	Any thrombosis	Deep vein thrombosis	Pulmonary embolism
Median time from fracture to surgery (h)	25	25	25	22.5
p-value		0.8	1	0.2
Median time from admission to surgery (h)	21	22	22	20
p-value		0.47	0.38	0.99
Type of anesthetic				
General	2,933 (98%)	75 (2.5%)	47 (1.6%)	29 (1.0%)
Spinal	1,837 (98%)	38 (2.0%)	29 (1.6%)	9 (0.5%)
Local	282 (99%)	2 (0.7%)	2 (0.7%)	0
p-value		0.12	0.53	0.05
Median length of anesthesia (min) **^a^**	60	60	60	60
p-value		0.05	0.09	0.3
Type of operation **^b^**				
Hemiarthroplasty	1,645 (98%)	28 (1.7%)	19 (1.1%)	9 (0.5%)
Sliding hip screw	1,863 (97%)	56 (2.9%)	40 (2.1%)	16 (0.8%)
Intramedullary nail	327 (97%)	11 (3.3%)	9 (2.7%)	2 (0.6%)
Multiple screws	1,262 (98%)	21 (1.6%)	11 (0.9%)	10 (0.8%)
Conservative	86 (99%)	1 (1.1%)	0 (0%)	1 (1.1%)
p-value		0.03	0.01	0.8

**^a^** Risk factor for any thrombosis significant at the 5% level. **^b^** Risk factor for any thrombosis significant at the 1% level.

The logistic regression analysis, adjusting for age and sex, showed that those patients who had lived in their own home and who had presented with an inter-trochanteric or sub-trochanteric fracture or with elevated hemoglobin at admission were at higher risk of developing a VTE. (Table 3) Other factors that were significant in univariate but not in multivariate analysis were: better preoperative mobility (p = 0.01), high mental test score (p = 0.005), and fixation with a dynamic hip screw (p = 0.03).

**Table 3.** Logistic regression adjusting for age and sex (incidence odds ratios of thrombosis for factors significant in multivariate analysis)

	No thrombosis	Any thrombosis	Odds ratio (95% CI)	p-value
Sex				
Female	4,062 (98%)	97 (2.3%)	1	0.09
Male	1,121 (98%)	20 (1.8%)	0.64 (0.38–1.07)	
Mean age (SD)	80 (11)	79 (12)	1.0 (0.98–1.02)	0.9
Residence on admission				
Own home	3,715 (97%)	99 (2.6%)	2.24 (1.32–3.82)	0.003
Institution	1,468 (99%)	18 (1.2%)	1	
Mean hemoglobin on admission (SD)	124 (17)	128 (18)	1.01 (1.0–1.03)	0.01
Type of fracture				
Intracapsular	2,917 (98%)	50 (1.7%)	1	0.001
Intertrochanteric	2,032 (97%)	63 (3.0%)	2.15 (1.46–3.17)	
Subtrochanteric	180 (98%)	4 (2.2%)	1.51 (0.53–4.30)	

**Figure 1.**
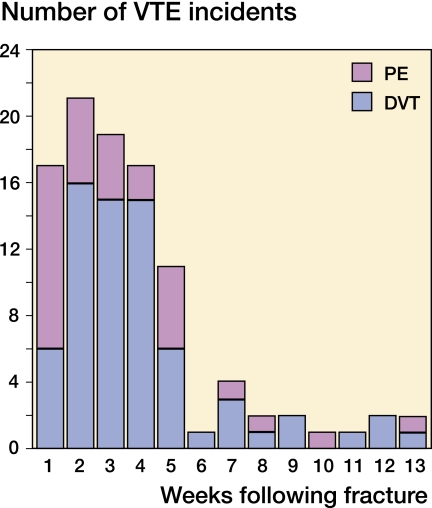
Time from fracture to diagnosis of thromboembolic complications.

The mean time to presentation and diagnosis of the VTE was 24 (3–91) days, with patients presenting up to 13 weeks after injury. (Figure 1) The mean time to presentation of deep vein thrombosis was 25 (4–91) days and the mean time to presentation of pulmonary embolism was 20 (3–81) days. 85% of these events occurred within 5 weeks of the fracture.

## Discussion

In this study of 5,300 hip fracture patients who received heparin thromboprophylaxis, we found an incidence of symptomatic VTE of 2.2%. This rate is in keeping with other reports, where symptomatic thromboembolism rates of between 1.3% and 6% have been reported (Todd et al. 1995, PEP 2000, Eriksson and Lasssen 2003, Rosencher et al. 2006). By comparison, most studies of VTE and its prevention have used sensitive diagnostic tests to detect deep vein thrombosis and, as such, would report a higher incidence of VTE. Thus, rates of VTE of up to 42% for patients with proximal femoral fractures have been reported (Handoll et al. 2002).

While patients with hip fractures are identified as belonging to a high risk group for development of thromboembolism (Geerts et al. 2004), it is difficult to predict which individual patients in a given risk group will develop a clinically important thromboembolic event. Our study suggests that patients with either inter-trochanteric or sub-trochanteric fractures, those living in their own home, and those admitted with an elevated hemoglobin value are at higher risk of development of symptomatic VTE.

Other authors have been unable to identify a difference in the incidence of thromboembolic complications between those patients with intracapsular fractures and those with extracapsular fractures (Hefley et al. 1996). We suggest that one reason for the difference in the rate of VTE between extracapsular and intracapsular fractures may be the more extensive blood loss within the tissues, and the resultant swelling of the limb that occurs with an extracapsular fracture.

Regarding the other risk factors, the reasons are not entirely clear. For those patients who are more mobile and living in their own home, it is possible that the lower mortality rate in this group of patients may be part of the explanation for the higher incidence of VTE; and it is also possible that these patients are more likely to report symptoms suggestive of thrombosis (Parker 2001).

We were not able to demonstrate any increase in the occurrence of VTE related to the delay of surgery (p = 0.8). This lack of any association has also been found in previous studies (Bredahl et al. 1992, Davis et al. 1998, Grimes et al. 2002, Moran et al. 2005). However, other studies have reported an association between surgical delay and an increased risk of VTE (Hefley et al. 1996, Zahn et al. 1999, Elder et al. 2005).

The univariate analysis demonstrated a statistically significantly higher number of PEs diagnosed in patients who had undergone surgery using a general anesthetic. Such significance was not apparent in the total number of VTE patients or in the subcategory of patients with deep vein thrombosis. While previous studies have indicated that the incidence of VTE may be reduced with spinal anesthesia (Parker et al. 2004), possibly due to an increased peripheral blood flow induced by spinal anesthesia, we were unable to demonstrate any statistically significant reduction in the occurrence of VTE for patients in our series.

We are aware of the weaknesses of our study. We had to use the ASA grade as a marker of co-morbidity at the time of operation. We found it impossible to analyze independent co-morbidities, such as heart failure, as the recording of past medical histories was often unreliable. However, we did find it surprising that there was no statistically significant increase in the incidence of VTE in those patients with higher ASA grades or in those with pathological fractures. In addition, it was not possible to analyze the data before and after the change in heparin regimes. However, a previous Cochrane review came to the conclusion that there was insufficient evidence to differentiate between the effectiveness of different heparin preparations (Handoll et al. 2002).

In summary, we found that the occurrence of clinically detected thrombosis after hip fracture was low in patients who are given low-dose subcutaneous heparin from admission. Despite thromboprophylaxis, various patient groups appear to be at a higher risk of developing a VTE. In these groups of patients, it may be appropriate to consider extended thromboprophylaxis.
